# Artificial Intelligence in Airway Management: Current Evidence and Future Perspectives

**DOI:** 10.7759/cureus.110566

**Published:** 2026-06-09

**Authors:** Nashrah Ashraf, Owais ul Umer Zargar

**Affiliations:** 1 Anesthesiology, Acharya Shri Chander College of Medical Sciences and Hospital, Jammu, IND; 2 General Surgery, Acharya Shri Chander College of Medical Sciences and Hospital, Jammu, IND

**Keywords:** airway management, artificial intelligence, deep learning, difficult airway, intubation, machine learning, medical education, ultrasound, unanticipated airway, videolaryngoscopy

## Abstract

Airway management remains a critical component of anesthetic practice, and failure to anticipate a difficult airway may result in significant morbidity and mortality. Conventional airway assessment tools demonstrate limited predictive accuracy and are often influenced by operator subjectivity. Recent advances in artificial intelligence (AI), particularly machine learning (ML) and deep learning (DL), have introduced novel approaches to airway assessment, prediction, procedural guidance, and education. This review aims to provide a comprehensive overview of the current applications of AI in airway management, evaluate the emerging evidence, discuss existing challenges, and explore future directions for clinical implementation. A narrative review of the literature was conducted using the PubMed, Scopus, and Google Scholar databases. Relevant studies, review articles, and guidelines published in English were screened to identify evidence related to AI-based airway assessment, difficult airway prediction, video laryngoscopy, airway imaging, simulation-based education, and emerging airway technologies.

AI has demonstrated promising applications across multiple domains of airway management. ML and DL models have shown improved performance in predicting difficult airways compared with conventional bedside assessment methods by incorporating clinical variables, facial image analysis, voice characteristics, and imaging data. AI-assisted ultrasound interpretation and videolaryngoscopy have enabled real-time anatomical recognition, procedural guidance, and automated performance assessment. Furthermore, AI-enhanced simulation and educational platforms have facilitated personalized training and objective competency evaluation. Despite these advances, challenges related to dataset quality, external validation, algorithm transparency, ethical considerations, and clinical integration remain significant barriers to widespread adoption. AI has the potential to transform airway management through enhanced prediction, decision support, procedural guidance, and education. While current evidence is encouraging, further multicenter studies, regulatory oversight, and the development of explainable AI systems are required before routine clinical implementation. AI should be considered a complementary tool that augments clinical expertise rather than a replacement for clinician judgment.

## Introduction and background

Airway management remains a cornerstone of anesthetic practice and is essential for ensuring adequate oxygenation and ventilation during surgical and critical care procedures. Failure to recognize and appropriately manage a difficult airway can result in severe complications, including hypoxaemia, aspiration, airway trauma, neurological injury, and death. Despite advances in airway devices and management algorithms, unanticipated difficult airway scenarios continue to contribute significantly to anesthesia-related morbidity and mortality [1]. Traditional bedside assessment methods, including the Mallampati classification, thyromental distance, upper lip bite test, and composite scoring systems, are widely used for preoperative airway evaluation. However, their predictive accuracy remains suboptimal, with considerable interobserver variability and limited sensitivity for identifying difficult airways [1,2].

Artificial intelligence (AI) has emerged as one of the most transformative technologies in modern healthcare. Using machine learning (ML), deep learning (DL), computer vision, and neural network algorithms, AI systems can analyze large volumes of clinical and imaging data, identify complex patterns, and generate predictive models with minimal human intervention. In anesthesiology, AI applications have expanded rapidly, encompassing perioperative risk prediction, hemodynamic monitoring, drug administration systems, and clinical decision support. More recently, growing interest has focused on the potential role of AI in airway management [3]. Recent studies have demonstrated promising results with facial image analysis, ultrasound-based assessment, voice analysis, and multimodal predictive models, often outperforming traditional airway assessment tools. Furthermore, advances in computer vision have facilitated the development of AI-assisted videolaryngoscopy systems capable of real-time anatomical recognition and procedural guidance [1-6].

Beyond airway prediction, AI has shown potential in airway education, simulation-based training, intelligent decision-support systems, and robotic-assisted airway interventions [4]. These innovations may enhance procedural accuracy, reduce operator-dependent variability, and improve patient safety. Nevertheless, several challenges remain, including limited external validation, concerns regarding algorithm transparency, data privacy, ethical considerations, and the need for robust clinical implementation studies [5]. AI tools are intended to augment, rather than replace, established guideline-based airway assessment and management strategies. This narrative review aims to summarize the current applications of AI in airway management, evaluate emerging evidence supporting its clinical use, discuss existing limitations and challenges, and explore future directions for integrating AI into airway assessment and management strategies [5,6].

## Review

Methods

A narrative literature review was conducted to evaluate the current applications of AI in airway management. A comprehensive search of PubMed, Scopus, and Google Scholar databases was performed to identify relevant studies published between January 2020 and March 2026. The final literature search was conducted in March 2026. The search strategy included combinations of the keywords “artificial intelligence”, “machine learning”, “deep learning”, “airway management”, “difficult airway”, “difficult intubation”, “difficult laryngoscopy”, “videolaryngoscopy”, “airway ultrasound”, “facial recognition”, “voice analysis”, and “airway education”. Reference lists of selected articles were also manually screened to identify additional relevant publications. Original research articles, systematic reviews, meta-analyses, narrative reviews, and relevant clinical guidelines published in the English language were considered for inclusion.

Studies focusing on AI-based difficult airway prediction, facial image analysis, voice-based assessment, ultrasound-assisted airway evaluation, videolaryngoscopy, simulation-based education, and emerging airway technologies were prioritized. Articles not related to airway management, non-English publications, conference abstracts without full-text availability, editorials lacking substantial scientific content, and studies with insufficient methodological detail were excluded. The selected literature was reviewed and synthesized narratively, with findings organized into major thematic domains including difficult airway prediction, videolaryngoscopy, education and simulation, challenges and limitations, and future directions of AI in airway management. As this study was designed as a narrative review, a formal risk-of-bias assessment was not performed; however, methodological limitations of the included literature, including dataset size, external validation, algorithm transparency, and potential bias, were considered during evidence synthesis.

Overview of artificial intelligence in airway management

AI refers to computer systems capable of performing tasks that traditionally require human intelligence, including learning, reasoning, problem-solving, pattern recognition, and decision-making. Recent advances in computational power, data availability, and algorithm development have accelerated the integration of AI into healthcare, enabling the analysis of complex clinical datasets and supporting evidence-based decision-making. In anesthesiology, AI has found applications in perioperative risk assessment, hemodynamic monitoring, drug delivery systems, operating room management, and airway management [6].

ML, a major subset of AI, enables computers to learn from data and improve their predictive performance without explicit programming. ML algorithms identify relationships among variables and generate predictive models that can be applied to previously unseen data. These models have demonstrated considerable utility in healthcare, particularly in risk prediction, outcome forecasting, and clinical decision-support systems. In airway management, ML algorithms can integrate multiple patient-related variables, including demographic characteristics, anthropometric measurements, medical history, and clinical examination findings, to predict difficult laryngoscopy and tracheal intubation more accurately than traditional bedside assessment methods [7,8]

DL, a more advanced subset of ML, utilizes multilayered artificial neural networks to process large and complex datasets. Unlike conventional ML algorithms that often rely on manually selected features, DL models can automatically identify and extract relevant features from raw data. Convolutional neural networks (CNNs), one of the most widely used DL architectures, have demonstrated exceptional performance in image analysis and computer vision tasks. These capabilities have facilitated the development of automated airway assessment systems using facial photographs, ultrasound images, computed tomography scans, magnetic resonance imaging, and videolaryngoscopy recordings [8].

The application of AI in airway management has evolved considerably over recent years. Early efforts primarily focused on improving the prediction of difficult airways through the analysis of conventional clinical parameters. More recently, AI-based systems have incorporated novel data sources such as facial morphology, voice characteristics, imaging studies, and real-time videolaryngoscopy images. These approaches aim to overcome the limitations of traditional airway assessment methods, which are often subjective, operator-dependent, and characterized by variable predictive accuracy [9].

Several studies have demonstrated promising results using AI-based predictive models. DL algorithms analyzing facial images have shown superior predictive performance compared with conventional bedside airway assessment tests. Similarly, ML models developed using multicentre clinical datasets have demonstrated improved discrimination in predicting difficult airway management and first-pass intubation success. Advances in ultrasound-based AI frameworks have further expanded the role of AI in airway assessment by enabling automated interpretation of airway anatomy and risk stratification [4-10]. The major clinical applications of AI in airway management are summarized in Table 1.

**Table 1:** Major applications of AI in airway management AI: artificial intelligence

Application area	AI technique	Clinical purpose	Potential benefits
Difficult airway prediction	Machine learning, deep learning	Predict difficult laryngoscopy and intubation	Improved risk stratification
Facial image analysis	Convolutional neural networks	Automated airway assessment	Non-invasive and rapid screening
Voice analysis	Machine learning algorithms	Identification of acoustic biomarkers	Simple and inexpensive assessment
Ultrasound-based assessment	Deep learning, computer vision	Automated interpretation of airway ultrasound	Reduced operator dependency
Videolaryngoscopy	Computer vision, deep learning	Real-time anatomical recognition and guidance	Improved intubation success
Education and simulation	AI-driven simulation systems	Skill development and assessment	Personalized training
Robotic airway management	AI-guided robotics	Assisted intubation procedures	Enhanced procedural precision

Artificial intelligence for difficult airway prediction

Accurate prediction of a difficult airway remains one of the most important goals of preoperative airway assessment [11,12]. Failure to identify patients at risk of difficult laryngoscopy or tracheal intubation may result in multiple intubation attempts, hypoxemia, aspiration, airway trauma, neurological injury, and even death. Although conventional bedside tests such as the Mallampati classification, thyromental distance, sternomental distance, upper lip bite test, and composite scoring systems are widely used in clinical practice, their predictive accuracy remains limited. Furthermore, these assessments are often subjective and may vary according to examiner experience and patient characteristics [13].

AI has emerged as a promising solution to overcome the limitations of traditional airway assessment methods. By integrating multiple clinical variables and analyzing complex relationships within large datasets, AI-based models can generate more objective and accurate predictions of difficult airway management. Recent advances in ML and DL have facilitated the development of predictive algorithms using demographic characteristics, clinical examination findings, facial morphology, imaging data, and voice characteristics [14,15].

Machine Learning-Based Prediction Models

ML algorithms have demonstrated significant potential in predicting difficult airway management. Unlike traditional statistical models, ML algorithms can analyze nonlinear relationships among variables and continuously improve their predictive performance through training on large datasets. [15-17] In a multicentre prospective observational study involving 10,741 patients undergoing emergency tracheal intubation across 13 emergency departments, Yamanaka et al. developed seven ML models using routinely collected patient and airway assessment data [4].

Most ML models demonstrated significantly higher discrimination ability than the modified LEMON criteria in predicting difficult airways. The ensemble learning model achieved the highest predictive performance, with a c-statistic of 0.74 compared with 0.62 for the modified LEMON criteria (p < 0.001). Similarly, ML models outperformed conventional logistic regression in predicting first-pass intubation success, with the ensemble model achieving a c-statistic of 0.81 versus 0.76 for the reference model. These findings highlight the potential role of AI-assisted clinical decision-making in airway management. (16)

Several predictive models have incorporated routinely available clinical variables such as age, sex, body mass index, neck mobility, Mallampati score, and airway examination findings. These models have consistently demonstrated improved sensitivity and specificity compared with conventional bedside tests. Such findings suggest that AI-based approaches may provide clinicians with more reliable risk stratification before airway intervention [2-8].

Facial Recognition and Deep Learning

Recent developments in computer vision have enabled the application of DL algorithms to facial image analysis for difficult airway prediction [17-19]. Facial morphology contains numerous anatomical features associated with difficult laryngoscopy, many of which may not be readily appreciated during routine clinical examination. Convolutional neural networks (CNNs), a form of DL architecture, can automatically identify and analyze these features from digital photographs [20,21].

Xia and colleagues developed a DL-based facial analysis model using neural networks to predict difficult videolaryngoscopy [3]. The model analyzed seven facial photographs from each patient and demonstrated significantly better predictive performance than traditional bedside airway assessment methods and established clinical scoring systems. Importantly, the facial recognition model achieved higher accuracy in identifying difficult videolaryngoscopy, supporting the potential role of AI-driven image analysis as a non-invasive screening tool. The growing availability of smartphone technology and digital imaging platforms further enhances the feasibility of facial recognition-based airway assessment. Future applications may allow rapid bedside risk prediction using simple facial photographs obtained during preoperative evaluation [21-23].

Voice Analysis and Acoustic Biomarkers

Voice analysis represents an emerging and innovative application of AI in airway assessment. As discussed by Naik et al., AI has been explored to predict difficult intubation by integrating subjective factors such as facial appearance, speech features, habitus, and other poorly recognized characteristics that may not be captured by conventional airway assessment methods [1]. The anatomical characteristics of the upper airway influence voice production and generate subtle acoustic patterns that may reflect airway anatomy. Sorbello et al. highlighted voice analysis as one of the most promising AI-based approaches for difficult airway prediction, allowing ML algorithms to identify acoustic biomarkers that are not readily apparent during routine clinical examination [7].

In a multicentre observational study involving 313 surgical patients, Rodiera et al. investigated the use of voice recordings combined with demographic variables to predict difficult airway management [11]. ML models incorporating acoustic parameters such as harmonics, shimmer, jitter, and harmonic-to-noise ratio demonstrated excellent predictive performance, achieving area under the receiver operating characteristic curve (AUC) values of 0.90 and 0.91. The authors concluded that acoustic voice parameters, when analyzed using ML algorithms, showed a promising ability to predict difficult airways preoperatively.

Similarly, Wilk et al. emphasized that AI-based airway assessment methods, including voice analysis, may overcome the limitations of traditional bedside tests by identifying complex relationships among anatomical and physiological variables, thereby improving prediction accuracy and supporting clinical decision-making [2]. Although further validation in larger and more diverse populations is required, current evidence suggests that voice-based prediction models may provide a simple, non-invasive, and readily accessible adjunct to conventional airway assessment techniques, potentially improving perioperative risk stratification and patient safety.

Ultrasound-Based Artificial Intelligence Models

Ultrasound has gained increasing popularity in airway assessment because of its ability to provide real-time visualization of upper airway anatomy. Traditional ultrasound-based airway evaluation relies on the measurement of anatomical structures such as the hyoid bone, epiglottis, vocal cords, and anterior neck soft tissues. However, as noted by Wang et al., ultrasound assessment remains highly operator dependent and requires substantial expertise for image acquisition and interpretation, limiting its widespread clinical application [5].

Recent advances in AI have enabled the integration of ultrasound imaging with ML and DL algorithms to improve the prediction of difficult airways. According to Naik et al., AI-based systems can analyze complex imaging data and identify anatomical patterns that may not be readily apparent during conventional airway assessment, thereby improving diagnostic accuracy and reducing subjective interpretation [1]. De Rosa et al. further highlighted that AI models combining imaging modalities with clinical assessment have the potential to enhance difficult airway prediction by recognizing complex anatomical relationships and generating more objective risk stratification tools. These approaches may provide clinicians with real-time decision support during airway evaluation and management [10].

More recently, Fu et al. developed an ultrasound-based AI framework for difficult airway prediction using a two-model, three-step hierarchical strategy [6]. Their study included 903 patients and utilized convolutional neural network algorithms to analyze ultrasound images obtained from multiple cervical planes. In the independent test set, the initial screening model achieved an AUC of 0.86 with sensitivity and specificity of 84%, while the secondary risk-stratification model achieved an AUC of 0.82. The authors concluded that ultrasound-based AI systems may serve as valuable clinical decision-support tools for risk stratification of difficult laryngoscopic exposure. Although further multicentre validation is required, current evidence suggests that ultrasound-based AI models may improve the objectivity, reproducibility, and diagnostic performance of airway assessment while reducing operator dependency. Such systems represent a promising advancement toward more standardized and accurate preoperative difficult airway prediction.

Artificial intelligence in videolaryngoscopy and real-time airway guidance

Videolaryngoscopy has transformed modern airway management by providing an indirect view of the glottis and improving first-pass intubation success rates compared with conventional direct laryngoscopy [21,22]. Despite these advantages, successful videolaryngoscopy remains dependent on operator experience, correct identification of anatomical landmarks, and appropriate device manipulation. Recent advances in AI, particularly computer vision and DL technologies, have opened new possibilities for enhancing videolaryngoscopy through real-time image analysis, procedural guidance, and automated performance assessment [23].

Real-Time Anatomical Recognition

One of the most promising applications of AI in videolaryngoscopy is the automated recognition of airway anatomy. Accurate identification of anatomical landmarks such as the epiglottis, vocal cords, arytenoid cartilages, and glottic opening is essential for successful tracheal intubation. However, visualization and recognition of these structures may be challenging in patients with distorted airway anatomy, obesity, airway pathology, or limited laryngeal exposure. As highlighted by Sorbello et al., AI-assisted videolaryngoscopy systems have the potential to provide real-time identification of airway structures and procedural guidance during intubation, thereby supporting clinicians during complex airway management scenarios [7].

Recent advances in DL and computer vision have enabled the development of algorithms capable of analyzing videolaryngoscopic images and automatically identifying key anatomical landmarks. Wilk et al. reported that DL models trained on large image datasets demonstrate high sensitivity and specificity in airway assessment and may significantly outperform traditional assessment methods in selected applications. These technologies allow continuous image analysis and real-time recognition of anatomical structures during airway interventions [2]. The clinical utility of such systems was demonstrated by Majeedi et al., who developed a DL model trained on 298,955 annotated frames from neonatal videolaryngoscopy recordings [8]. The model successfully identified optimal laryngoscope insertion depth and detected glottic-zone visualization with an F1 score of 0.894. The authors suggested that AI-enabled videolaryngoscopy may enhance anatomical recognition, procedural guidance, and operator training during airway management.

Similarly, De Rosa et al. emphasized that AI integrated with videolaryngoscopic imaging could provide real-time clinical decision support by recognizing complex anatomical patterns and assisting clinicians in difficult airway situations [10]. Such systems may be particularly beneficial for trainees and less experienced operators by facilitating rapid recognition of airway landmarks and reducing the likelihood of failed intubation attempts. Although further validation in diverse clinical settings is required, current evidence suggests that AI-assisted anatomical recognition may enhance the safety, efficiency, and success of videolaryngoscopy-guided intubation.

AI-Assisted Procedural Guidance

Beyond anatomical recognition, AI systems have the potential to provide procedural guidance during tracheal intubation. As discussed by Naik et al., AI applications in airway management extend beyond airway assessment and may support clinicians throughout the intubation process by analyzing visual, anatomical, and procedural data in real time [1]. Such systems are designed to improve patient safety and optimize airway management by providing objective decision support during critical procedures. ML algorithms can analyze laryngoscope position, blade movement, insertion depth, and the quality of glottic visualization during videolaryngoscopy. According to Sorbello et al., AI-powered videolaryngoscopy systems are capable of providing real-time procedural guidance, tracheal tube placement verification, and cognitive support during airway management, thereby helping clinicians maintain adherence to best-practice airway strategies [7].

Recent studies have demonstrated the feasibility of DL models capable of analyzing videolaryngoscopy recordings and identifying optimal procedural techniques. In a study by Majeedi et al., a DL model trained on 298,955 annotated videolaryngoscopy frames successfully classified laryngoscope insertion depth as shallow, glottic zone, or deep. The model identified optimal glottic-zone insertion with an F1 score of 0.894, suggesting that AI-enabled systems may provide immediate feedback regarding blade advancement and positioning during intubation. The authors proposed that such technologies could support both clinical practice and procedural training [8].

Similarly, De Rosa et al. highlighted the potential of AI-driven imaging systems to provide real-time clinical decision support by integrating anatomical recognition with procedural guidance. These technologies may assist operators in achieving optimal glottic exposure, reducing unnecessary manipulation, and improving first-pass intubation success rates [10]. Future developments may enable AI-assisted videolaryngoscopes to function as intelligent navigation systems that continuously analyze procedural performance and provide dynamic recommendations throughout airway management.

Automated Verification of Tracheal Intubation

Incorrect endotracheal tube placement remains one of the most serious complications of airway management and may result in hypoxemia, aspiration, cardiac arrest, or death if not promptly recognized [21]. Accurate and rapid confirmation of tracheal intubation is therefore essential for patient safety. Recent advances in AI have led to the development of image-recognition systems capable of assisting clinicians in verifying correct tube placement using videolaryngoscopy and other imaging modalities [14]. 

According to Sorbello et al., AI-powered videolaryngoscopy systems have demonstrated the ability to provide real-time tracheal tube placement verification in addition to anatomical recognition and procedural guidance [7]. Such systems continuously analyze visual information during intubation and may support clinicians in confirming successful airway placement while reducing the risk of human error. Similarly, De Rosa et al. highlighted the growing role of AI in airway imaging and clinical decision support. By integrating computer vision algorithms with videolaryngoscopic imaging, AI systems can identify airway landmarks and evaluate the relationship between the endotracheal tube and surrounding anatomical structures, thereby facilitating automated confirmation of successful tracheal intubation [10].

Recent developments in DL have demonstrated that computer vision models can accurately analyze airway images and recognize clinically relevant patterns in real time. Wilk et al. reported that AI-based airway assessment systems have shown high diagnostic performance and may significantly improve the accuracy and objectivity of airway-related decision-making [2]. These capabilities provide the foundation for automated tube-placement verification systems that can rapidly identify successful tracheal intubation and potentially alert clinicians to oesophageal placement or other airway complications.

Although further prospective validation studies are required before widespread implementation, current evidence suggests that AI-assisted verification systems have the potential to improve patient safety, reduce airway-related complications, and serve as an additional safeguard during tracheal intubation.

Performance Assessment and Training

Videolaryngoscopy provides a unique opportunity for objective assessment of airway management skills because the entire procedure can be recorded and analyzed [24-28]. AI algorithms can evaluate multiple aspects of performance, including time to glottic visualization, quality of laryngeal exposure, blade handling, tube advancement, and overall procedural efficiency [28]. Automated performance assessment systems may be integrated into simulation-based education and clinical training programmes. Such tools can provide personalized feedback to trainees, identify technical deficiencies, and monitor skill progression over time. Compared with traditional instructor-based assessment, AI-driven evaluation offers greater objectivity and consistency while reducing the educational burden on faculty members [27].

Artificial intelligence in airway education and simulation

Airway management is a fundamental skill in anesthesiology, emergency medicine, and critical care. Achieving competence in airway assessment and tracheal intubation requires extensive training, repeated practice, and continuous performance evaluation. Traditional airway education relies on didactic teaching, mannequin-based simulation, supervised clinical experience, and instructor feedback. While these methods remain essential, they may be limited by variability in teaching quality, restricted access to expert supervision, and difficulties in providing objective performance assessment. Recent advances in AI offer new opportunities to enhance airway education through intelligent simulation systems, automated feedback mechanisms, and personalized learning platforms [14-20].

AI has increasingly been integrated into medical education, particularly in simulation-based training environments. ML and DL algorithms can analyze trainee performance, identify technical errors, and provide real-time feedback. Such systems allow learners to practice airway management skills repeatedly in a risk-free environment while receiving objective assessments of their performance.

Automated Performance Assessment

One of the major advantages of AI in medical education is its ability to provide objective and consistent performance evaluation. Traditional assessment often depends on subjective instructor observations, which may vary between assessors. In contrast, AI systems can analyze procedural metrics with high precision [26]. During airway simulation, ML algorithms can evaluate parameters such as time to intubation, laryngoscope handling, glottic visualization, tube placement accuracy, and adherence to airway management algorithms. These data can be used to generate detailed performance reports highlighting strengths and areas requiring improvement. Automated assessment allows trainees to receive immediate feedback and facilitates continuous monitoring of skill development [29]. Furthermore, AI-driven evaluation systems may reduce faculty workload by automating routine assessments while maintaining educational quality. Such tools may be particularly useful in institutions with limited access to expert airway instructors.

Virtual Reality and Augmented Reality Applications

Virtual reality technologies have gained popularity in airway management training because they provide immersive and interactive learning experiences. AI-enhanced VR systems can simulate a wide range of clinical scenarios, including difficult airway management, emergency intubation, and surgical airway procedures [29]. By integrating computer vision and ML algorithms, virtual reality platforms can analyze user actions in real time and provide corrective guidance during training exercises. Augmented reality systems may further enhance learning by overlaying anatomical structures, procedural instructions, or performance indicators onto the learner’s visual field. Such technologies can improve spatial understanding of airway anatomy and facilitate the development of procedural skills. Key studies evaluating AI-based airway assessment and prediction models are summarized in Table 2.

**Table 2:** Key studies evaluating AI in airway management AI: artificial intelligence; ML: machine learning; LEMON: look, evaluate, Mallampati, obstruction, neck mobility

Author	Year	AI method	Study focus	Main findings
Yamanaka et al. [4]	2022	ML	Difficult airway prediction and first-pass success	ML models outperformed the modified LEMON criteria
Xia et al. [3]	2024	Deep learning facial analysis	Difficult videolaryngoscopy prediction	The facial recognition model performed better than traditional assessments
Rodiera et al. [11]	2024	Machine learning	Voice-based airway prediction	Voice characteristics predicted a difficult airway with high accuracy
Wilk et al. [2]	2025	Review article	AI in difficult airway assessment	AI models showed superior sensitivity and specificity compared with traditional tests
Alatau et al. [9]	2025	Review article	AI prediction of difficult airways	Facial image-based deep learning models demonstrated the highest predictive performance
Fu et al. [6]	2026	Ultrasound-based AI framework	Difficult airway risk stratification	AI-assisted ultrasound showed promising diagnostic accuracy
Sorbello et al. [7]	2026	Narrative review	AI applications in airway management	Highlighted roles in prediction, videolaryngoscopy, education, and robotics

Future directions

AI is expected to play an increasingly important role in airway management as computational capabilities, data availability, and algorithm performance continue to improve. Although current applications are primarily focused on difficult airway prediction and procedural support, future developments may transform multiple aspects of airway assessment, decision-making, training, and clinical practice. One of the most promising future applications is the development of multimodal predictive systems that integrate diverse sources of patient information. Current airway assessment methods often rely on individual clinical parameters or isolated imaging findings. Future AI platforms may combine demographic characteristics, facial photographs, voice recordings, ultrasound images, radiological data, electronic health records, and physiological measurements into a single predictive framework. Such comprehensive models could provide highly personalized risk assessments and improve the identification of patients at risk for difficult airway management. [19-21]

Advances in computer vision and DL are also likely to enhance real-time airway guidance during videolaryngoscopy and tracheal intubation. Future AI-enabled videolaryngoscopes may automatically identify airway structures, assess glottic visualization, verify endotracheal tube placement, and provide immediate procedural recommendations. Integration of augmented reality technology may allow anatomical landmarks and guidance prompts to be projected directly onto the operator’s display, potentially improving first-pass intubation success and reducing complications. [22-24]

Ultrasound-based airway assessment represents another area of significant future potential. Automated image interpretation systems may simplify airway ultrasonography and reduce operator dependency. AI algorithms capable of real-time analysis of airway anatomy could facilitate rapid bedside assessment, particularly in emergency and critical care settings. As portable ultrasound devices become increasingly available, integration with AI may expand access to advanced airway evaluation tools. [26] AI may also contribute to the development of intelligent clinical decision-support systems. Such platforms could assist clinicians in selecting appropriate airway management strategies based on patient-specific characteristics and current clinical guidelines. During high-stress situations, AI systems may function as cognitive aids by providing reminders, risk alerts, and evidence-based recommendations. These tools have the potential to reduce human error and improve adherence to established airway management algorithms.

The future may also witness the emergence of robotic and semi-autonomous airway management systems. Experimental studies have already demonstrated the feasibility of AI-guided robotic intubation under controlled conditions. Although widespread clinical implementation remains distant, advances in robotics, ML, and computer vision may eventually enable remote airway interventions and support airway management in resource-limited environments. In medical education, AI-powered simulation platforms are expected to become increasingly sophisticated. Adaptive learning systems capable of tailoring educational content to individual learner needs may improve training efficiency and competency development. Virtual reality and augmented reality technologies integrated with AI-driven feedback systems may provide highly realistic and personalized learning experiences for airway management trainees.

Despite these exciting possibilities, future progress will depend on addressing several important challenges. Large multicentre studies are needed to validate existing models across diverse populations and clinical settings. The development of explainable AI frameworks will be essential to improve transparency and clinician trust. Regulatory oversight, ethical governance, and robust data security measures must evolve alongside technological advances to ensure safe and responsible implementation. Ultimately, the future of AI in airway management is unlikely to involve the replacement of clinicians. Instead, AI is expected to function as a powerful adjunct that complements human expertise by enhancing prediction, improving decision-making, supporting procedural performance, and facilitating education. The most effective airway management strategies will likely arise from collaboration between skilled clinicians and intelligent technological systems. Emerging applications and future directions of AI in airway management are summarized in Table 3.

**Table 3:** Future directions of AI in airway management AI: artificial intelligence

Emerging area	Potential clinical application
Multimodal AI models	Integration of facial images, voice analysis, ultrasound, and clinical data for comprehensive airway prediction
AI-assisted videolaryngoscopy	Real-time anatomical recognition and procedural guidance
Automated ultrasound interpretation	Objective and rapid airway assessment
Augmented reality-guided airway management	Visual overlay of airway structures during procedures
Intelligent clinical decision-support systems	Personalized airway management recommendations
AI-powered simulation and education	Adaptive training and competency assessment
Robotic-assisted airway interventions	Precision-guided and remote airway management
Explainable AI platforms	Improved transparency and clinician trust

Challenges and limitations of artificial intelligence in airway management

Despite the promising advancements in AI for airway assessment, prediction, videolaryngoscopy, and medical education, several challenges continue to limit its widespread clinical implementation. While many AI-based models have demonstrated encouraging diagnostic performance under research conditions, translating these technologies into routine clinical practice remains a complex process.

Limited Dataset Size and Generalizability

The performance of AI algorithms is highly dependent on the quality, quantity, and diversity of training data. Many currently available airway prediction models have been developed using relatively small datasets obtained from single institutions. Such datasets may not adequately represent the broad range of patient characteristics encountered in clinical practice. Variations in ethnicity, age, body habitus, craniofacial anatomy, and comorbid conditions may influence model performance when applied to external populations. Furthermore, most studies evaluating AI-based airway assessment have focused on elective surgical patients. Consequently, the applicability of these models in emergency departments, intensive care units, pediatric populations, and patients with abnormal airway anatomy remains uncertain. Multicentre studies involving diverse patient populations are required to improve the generalizability of AI systems.

Lack of External Validation

Although numerous ML and DL models have demonstrated high predictive accuracy, many have undergone only internal validation. External validation using independent datasets is essential to establish reliability and reproducibility across different clinical settings. Without rigorous validation, the risk of overfitting remains substantial, whereby a model performs exceptionally well on training data but poorly in real-world clinical practice. The absence of standardized evaluation criteria further complicates comparisons among studies. Differences in outcome definitions, airway assessment methods, imaging protocols, and statistical reporting make it difficult to determine which AI models are most suitable for clinical adoption.

Algorithm Transparency and Explainability

One of the most frequently cited concerns regarding AI in healthcare is the “black-box” nature of many ML and DL algorithms. Complex neural networks often generate predictions without providing clear explanations regarding how decisions are reached. This lack of transparency may reduce clinician confidence and create challenges in high-stakes clinical situations. In airway management, clinicians must understand the rationale behind risk predictions before modifying management plans. Explainable AI (XAI) has emerged as a potential solution by providing interpretable outputs that identify factors contributing to algorithmic decisions. Future airway prediction systems should prioritize explainability to improve clinician trust and facilitate clinical integration.

Ethical and Legal Considerations

The increasing use of AI in healthcare raises important ethical and legal questions. AI systems frequently require large amounts of patient data, including clinical information, facial photographs, voice recordings, ultrasound images, and videolaryngoscopy recordings. Ensuring patient confidentiality and data security is therefore essential. Additionally, uncertainty exists regarding legal responsibility when AI-assisted recommendations contribute to adverse outcomes. Questions concerning liability among clinicians, healthcare institutions, and technology developers remain unresolved. The establishment of clear regulatory frameworks will be critical as AI becomes increasingly integrated into clinical practice.

Bias and Equity Concerns

Algorithmic bias represents another important challenge. AI models trained on datasets that inadequately represent certain populations may demonstrate reduced accuracy in underrepresented groups. Such biases may inadvertently contribute to healthcare disparities and unequal clinical outcomes. For example, facial recognition algorithms developed using predominantly homogeneous populations may perform less effectively in individuals with different ethnic or anatomical characteristics. Addressing these concerns requires the inclusion of diverse patient populations during model development and validation.

Integration into Clinical Workflow

Successful implementation of AI systems requires seamless integration into existing clinical workflows. Complex software interfaces, additional equipment requirements, and increased documentation burdens may reduce clinician acceptance. AI tools should therefore be designed to complement clinical practice without increasing workload or disrupting established airway management processes. Training healthcare professionals to appropriately interpret and utilize AI-generated recommendations will also be essential. Clinicians must understand both the capabilities and limitations of AI systems to ensure safe and effective implementation.

Risk of Overreliance and Deskilling

Although AI can provide valuable decision support, excessive reliance on automated systems may result in reduced development or maintenance of clinical skills. Airway management often requires rapid adaptation to unexpected situations that may not be adequately addressed by algorithmic recommendations. Clinical judgement, situational awareness, communication, and technical expertise remain indispensable components of safe airway management. Therefore, AI should be viewed as a tool that augments human performance rather than replacing clinician expertise. Maintaining an appropriate balance between technological assistance and independent clinical decision-making will be critical as AI technologies continue to evolve.

In summary, despite substantial progress in the application of AI to airway management, important challenges related to validation, transparency, ethics, bias, and clinical integration remain. Addressing these limitations through collaborative research, robust regulatory oversight, and multidisciplinary development efforts will be essential for the safe and effective adoption of AI-based airway technologies. The principal challenges and limitations affecting clinical implementation of AI in airway management are outlined in Table 4.

**Table 4:** Challenges and limitations of AI in airway management AI: artificial intelligence

Challenge	Description	Potential solution
Limited dataset size	Many AI models are developed using single-centre datasets with limited population diversity	Large multicentre collaborative databases
Lack of external validation	Performance may decrease when applied to different patient populations	Prospective multicentre validation studies
Algorithm transparency	Deep learning models often function as “black boxes”	Development of explainable AI (XAI) systems
Data privacy and security	Use of facial images, voice recordings, and clinical data raises confidentiality concerns	Robust data protection and regulatory frameworks
Algorithmic bias	Underrepresentation of certain populations may affect prediction accuracy	Diverse and representative training datasets
Integration into clinical workflow	Additional hardware and software requirements may limit adoption	User-friendly interfaces and seamless integration
Regulatory and legal issues	Uncertainty regarding accountability for AI-assisted decisions	Clear regulatory guidelines and legal frameworks
Risk of overreliance on AI	Excessive dependence may contribute to clinician deskilling	Maintain human oversight and regular training

## Conclusions

AI has emerged as a promising technology with the potential to transform multiple aspects of airway management. Applications of ML and DL have demonstrated encouraging results in difficult airway prediction, facial image analysis, voice-based assessment, ultrasound interpretation, videolaryngoscopy guidance, and simulation-based education. Compared with traditional airway assessment methods, AI-based approaches offer the potential for greater objectivity, improved predictive accuracy, and enhanced clinical decision support. Despite these advances, important limitations remain, including limited dataset diversity, limited external validation, concerns about algorithm transparency, and ethical and regulatory challenges. Current evidence supports the role of AI as a complementary tool rather than a replacement for clinical expertise. Human judgement, technical skills, and situational awareness remain essential components of safe airway management.
